# Migration intra-péritonéal d'un dispositif intra utérin diagnostiqué 20 ans après l'insertion: à propos d'un cas

**DOI:** 10.11604/pamj.2014.17.7.3790

**Published:** 2014-01-13

**Authors:** Wail Bouzouba, Fatime Zahra Fdili Alaoui, Sofia Jayi, Hakima Bouguern, Moulay Abdelilah Melhouf

**Affiliations:** 1 Service de Gynecologie Obstetrique II, Chu Hassan II, Fes, Maroc

**Keywords:** DIU, migration intra-péritonéale, coelioscopie, IUD, intraperitoneal migration, laparoscopy

## Abstract

Le dispositif intra-utérin (DIU) est une des méthodes contraceptives les plus efficaces et les plus utilisé à travers le monde: environ 100 millions d'utilisatrices. La perforation reste exceptionnelle âpres la pose d'un DIU cependant c'est une des complications les plus graves. Nous rapportant le cas d'une patiente de 49 ans, notion de pose de stérilet il y a 20 ans, suivie en oncologie pour un carcinome canalaire infiltrant du sein ayant bénéficiée d'un patey puis chimiothérapie adjuvante actuellement sous hormonothérapie, qui dans le cadre du bilan d'extension, un scanner thoraco-abdomino-pelvienne a objectivé la présence d'un DIU en sous hépatique. Sous guidage coelioscopique, on a réussie à retirer le DIU qui était enchâssé dans l’épiploon au niveau de la gouttière pariéto-colique droite. Nous insistons à travers cette observation et sous la lumière de la revue de la littérature sur l'efficacité et l'innocuité du DIU lorsque la technique et les indications sont rigoureusement respectées, mais aussi sur une des complications rarissime de la pose du DIU, et à mettre en évidence le rôle diagnostic et thérapeutique de la cœlioscopie dans la prise en charge de ces migrations.

## Introduction

La contraception par dispositif intra-utérin est l'une des plus utilisée au monde, environ 100 millions d'utilisatrices, il s'agit d'une méthode simple, efficace et réversible avec un indice de Pearl inférieur à 1 pour 100 années femme. Son mode d'action contraceptif se situe au niveau des trompes et des spermatozoïdes, ainsi qu'au niveau de la cavité utérine. Cependant, leurs effets secondaires, ainsi que leurs complications et contre-indications, doivent être connus pour optimiser son action [1, 2]. La perforation est l'une des complications les plus rares et les plus graves, et qui peut engendrer la migration du DIU dans les différents organes de voisinage. Il a été décrit des migrations au niveau du cul-de-sac du Douglas, au niveau de l’épiploon, du mésentère, du colon et au niveau de la vessie [2]. Nous rapportons un nouveau cas de migration du DIU dans la cavité péritonéal, dont le diagnostic a été fait 20 ans après la pose et dans le cadre du bilan d'extension d'un cancer du sein. L'abdomen sans préparation et la tomodensitométrie étaient les moyens de diagnostic de cette migration, la c'lioscopie a permis de retirer le DIU qui était enchâssé dans l’épiploon.

## Patient et observation

**Figure 1:**
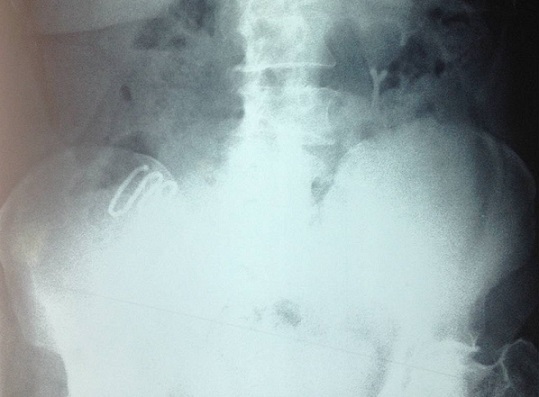
L'ASP en position debout avec un DIU type boucle de Lippes en sous hépatique

**Figure 2:**
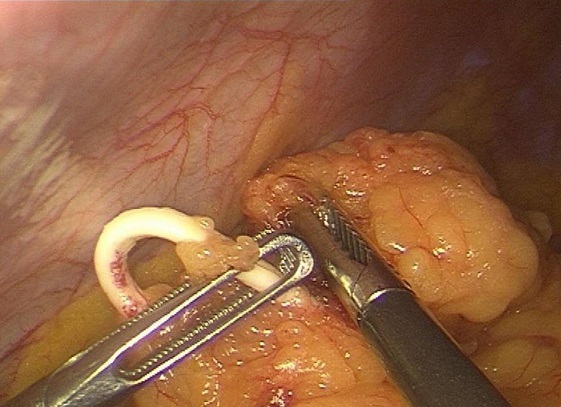
Image coelioscopique du DIU type boucle de Lippes enchâssé dans l’épiploon

**Figure 3:**
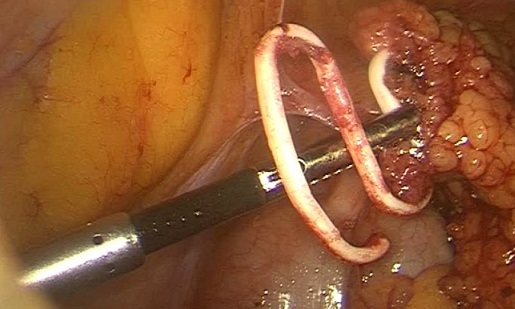
Image coelioscopique du DIU type boucle de Lippes enchâssé dans l’épiploon, au niveau de la gouttière pariéto-colique droite

Il s'agit de madame M.Z âgée de 49 ans, multipare, ayant eu ses accouchements par voie basse et porteuse d'un DIU depuis 20 ans, qui a été retiré d'après la patiente 4 mois après la pose, suivie en oncologie pour un carcinome canalaire infiltrant du sein droit depuis 3 ans, ayant bénéficié d'un patey droit suivie de chimiothérapie, et qui est actuellement sous hormonothérapie. Admise dans notre service pour prise en charge d'une migration du DIU découvert après la réalisation d'un scanner thoraco-abdomino-pelvienne dans le cadre du bilan d'extension du cancer du sein. Sur le plan clinique la patiente ne présentait aucune symptomatologie abdomino-pelvienne ou gynécologique. L'examen clinique était sans particularité et n'a pas mis en évidence le fils du stérilet en intra-vaginal. Le scanner abdominale a objectivé le DIU en sous hépatique. L’échographie pelvienne ne retrouve pas le stérilet en intra-utérin. L'ASP (abdomen sans préparation) en position debout a confirmé la présence du DIU type boucle de Lippes dans la cavité abdominale, en sous hépatique (Figure 1). Le diagnostic de perforation secondaire de l'utérus avec migration en intra-abdominale est retenue. L'ablation par c'lioscopie est indiquée dans ce cas. L'exploration coelioscopique a objectivé la présence d'un DIU type boucle de Lippes enchâssé dans l’épiploon (Figure 2), au niveau de la gouttière pariéto-colique droite (Figure 3), qui a été retiré sans incident après son décollement de l’épiploon. La patiente a été revue un mois puis 3 mois après, elle est bien portante.

## Discussion

Le dispositif intra-utérin est la méthode de contraception la plus utilisée dans le monde, environ 100 millions d'utilisatrices, c′est une méthode contraceptive qui fait appel à un procédé mécanique d'action locale. Il existe actuellement plusieurs types de stérilets, les stérilets inertes (Boucle de Lippes) qui ne sont plus utilisées, et les stérilets bio-actifs, en cuivre, cuivre-argent ou progestatifs sont les plus utilisés en raison de leur meilleure tolérance [1].

L'insertion du DIU est un acte médical simple, régie par des obligations légales et des lois, nécessite un minimum de connaissance médicale et le minimum de pratique car dans certaines situations, cette insertion peut être suivie par des complications tel que l′infection ou la perforation utérine [1, 2]. L'incidence de la perforation est rare, elle ne dépasse pas 1,3 pour 1000 poses, selon de grands essais cliniques rapportés [3–5]. Ces perforations peuvent être partielles, quand une partie seulement du DIU perce la paroi de l'utérus ou le col, ou complètes, quand le DIU traverse la paroi de l'utérus pour pénétrer dans la cavité abdominale [2–5]. Elle se produit le plus souvent au moment de la pose, mais elle peut passer inaperçue et n’être découverte que secondairement [6]. Plusieurs facteurs peuvent intervenir et être à l'origine de la perforation, tout d'abord des facteurs utérins, avec une petite taille, une malposition importante, en particulier une rétroversion, une fragilité du myomètre par des grossesses multiples, les utérus hypoplasiques, les utérus cicatriciels, des facteurs liés à l'insertion en particulier qui nécessite une poussée, et l'inexpérience ou la maladresse de l'opérateur [1, 2]. Sur le plan physio-pathologique l'importance de l'inflammation endométriale qu'entraîne ce corps étranger empêche la nidation. Cette inflammation est une arme à double tranchant, entraînent une accumulation non négligeable d'enzymes et de substances lytiques lysosomiales favorisant la destruction endométriale et la migration du DIU [7]. Après perforation, le dispositif peut et dans la majorité des cas rester dans la cavité pelvienne, migrer dans les organes creux, notamment dans la vessie, ou s'entourer par l’épiploon et rester inerte pendant plusieurs années surtout pour les DIU non actif [8, 9], comme c'est le cas de notre patiente, qui 20 ans après la pose du stérilet un scanner abdominal dans le cadre du bilan d'extension d'un cancer du sein a objectivé la présence d′un DIU en sous hépatique. Dans la littérature nous avons dénombré plus de 120 cas de migration intra abdominal du DIU, 59 cas de migration intra-vésicale; les migrations pelviennes extra-vésicales et abdominales sont exceptionnelles [2–10].

Sur le plan clinique la symptomatologie est variable en fonction du siège de la migration et du type de stérilet, dans notre cas un stérilet inerte type boucle de lippes incrusté dans l′épiploon n’à causer aucune réaction inflammatoire ou autres, et il est resté asymptomatique pendant 20 ans, ce qui rejoins les résultats de la littérature car 85% des cas déclarés de perforation n′ont pas causés de complications et étaient asymptomatique au moment du diagnostic [9]. Mais dans certains cas le diagnostic peut se faire par l′apparition de signes cliniques types fièvre, douleurs abdominaux, diarrhées ou infections urinaires, mais aussi par l′apparition de complications tel qu′un syndrome occlusif, une péritonite par perforation d′un organe creux, et c′est le cas d′une patiente de 64 ans qui a présentée un syndrome occlusif par strangulation 31 ans après la pose d′un stérilet type Saf-T-Coil [9]. Ainsi la perforation utérine par DIU est habituellement asymptomatique. Sauf lorsqu'elle est concomitante à la pose, entraînant une douleur violente, qui doit attirer l'attention du médecin. A l'examen, la perforation est suspectée devant la disparition des fils repères, après s’être assuré que les fils ne sont pas remontés dans l'endocol, et c′est le cas de notre patiente, et chez qui le diagnostic a été fait d'une façon fortuite lors d'un examen radiologique pour une autre raison: bilan d'extension d'un cancer du sein [2–9]. Le diagnostic clinique n'est pas toujours évident, il doit faire appel à des explorations complémentaires pour localiser le dispositif.

La radiographie de l'abdomen sans préparation, après avoir éliminé une grossesse, confirme l'expulsion si le DIU n'est pas retrouvé sur le cliché. Sa visualisation ne préjuge en rien de sa situation [9]. Dans notre cas l′ASP a permis de confirmer l′existence du dispositif en sous hépatiques et d′avoir une idée sur son type. L’échographie sus-pubienne représente une étape fondamentale pour le diagnostic. Elle permet de visualiser ou non le stérilet en intra-utérin, ou dans une autre localisation [2–9]. Dans les cas ou le diagnostic paraclinique est difficile, une coelioscopie ou une laparotomie diagnostique peuvent avoir une place capitale.

A distance de la pose, l'indication du retrait d'un stérilet est impérative et doit être rapidement réalisée en raison des complications sus citées qui augmentent le risque de morbidité et de mortalité féminine [9]. Le traitement des migrations intra-abdominales reste le retrait par coelioscopie ou par laparotomie [9, 10]. Dans notre cas une coelioscopie a été réaliser et qui a permis de mettre en évidence un DIU type boucle de Lippes enchâssé dans l’épiploon au niveau de la gouttière pariéto-colique droite et qui a été retiré sans incident après son décollement de l’épiploon.

## Conclusion

Le stérilet est une méthode contraceptive efficace, son insertion est un acte médical simple qui nécessite un minimum de connaissances et d'expériences. La perforation est l'une des complications les plus rares et les plus graves. La coelioscopie reste le moyen diagnostic et thérapeutique le plus efficace.

## References

[1] Boudineau M, Multon O, Lopes P (2001). Contraception par dispositif intra-utérin. Encycl Méd Chir- Gynécologie.

[2] Zouhal A, el Amrani N, Bensaid F (2000-2001). Migration intra-vesicale d′un dispositif intra-uterin a propos d′un cas.

[3] Treiman K, Laurie Liskin SCM, Adrienne Kols (1995). Les DIU: état récent des informations. Population Reports (Series B).

[4] Ledward RS, Healey C, Eadie R (1972). Removal of extrauterine Saf-T-Coil through laparoscope. British Medical Journal..

[5] Zakin D, Stern WZ, Rosenblatt R (1981). Complete and partial uterine perforation and embedding following insertion of intrauterine devices I Classification, complications, mechanism, incidence, and missing string. Obstetrical & Gynecological Survey..

[6] Gruber A, Rabinerson D, Kaplan B, Pardo J, Neri A (1996). The missing forgotten intrauterine contraception device. Contraception..

[7] Chang CH, Chou CY, Lee WI, Tzeng CC, Liuc H (1992). Pelvic actinomycosis with colo-ileo-vesical fistula formation: report of case. J Formos Med Assoc..

[8] Markovitch O, Klein Z, Gidoni Y, Holzinger M, Beyth Y (2002). Extrauterine mislocated IUD: is surgical removal mandatory. Contraception..

[9] Brar R, Doddi S, Ramasamy A, Sinha P (2010). A forgotten migrated intrauterine contraceptive device is not always innocent: a case report. Case Rep Med..

[10] Bacha Khaled, Ben Amna Marouane, Ben Hassine Lofti (2001). Dispositif intra-utérin migré dans la vessie. Progrès en Urologie.

